# Effects of splenectomy and GTS-21, a selective α7 nicotinic acetylcholine receptor agonist, on the development of septic ileus in mice

**DOI:** 10.1186/cc14043

**Published:** 2014-12-03

**Authors:** S Nullens, JG De Man, PA Pelckmans, BY De Winter

**Affiliations:** 1 Laboratory for Experimental Medicine and Pediatrics, Division of Gastroenterology and Hepatology, University of Antwerp, Belgium; 2 Department of Gastroenterology and Hepatology, Antwerp University Hospital, Antwerp, Belgium

## Introduction

Sepsis remains a leading cause of mortality in our ICUs. Ileus, defined as the inhibition of the propulsive motility in the gastrointestinal (GI) tract, together with mucosal barrier dysfunction will maintain sepsis by the translocation of intestinal bacteria. Preliminary data in our group showed that the administration of GTS-21, a selective alpha7 nicotinic acetylcholine receptor (α7nAChR) agonist, ameliorates inflammation and GI motility disturbances during sepsis [1]. Activation of the α7nAChR is the final step in the vagal anti-inflammatory pathway, a spleen-dependent and macrophage-dependent pathway that dampens inflammation. We aimed to study the effects of splenectomy (SPLX) combined with GTS-21 on GI motility and on local colonic and systemic inflammation, as the role of the spleen in the vagal anti-inflammatory pathway is currently under debate.

## Methods

Septic ileus was induced in Swiss-OF 1 mice by cecal ligation and puncture (CLP). One week prior to CLP, mice underwent SPLX or sham surgical incision (sham). GTS-21 (8 mg/kg) or vehicle was administered intraperitoneally 1 hour before CLP, and once every 24 hours after the procedure. Mice were sacrificed 48 hours following CLP. Motility was assessed by measurement of GI transit of beads, with calculation of percentage of gastric emptying (%GE) and geometric center (GC). Serum samples were analyzed with cytometric bead array (CBA) for the proinflammatory cytokines IL-6 and TNFα. Supernatants of centrifuged homogenized colonic samples were assessed with CBA for IL-6 and TNFα.

## Results

Administration of GTS-21 resulted in a significant improvement in GI motility in septic mice, as was demonstrated by an increase in the %GE and GC (Table 1). GTS-21 significantly decreased the serum concentration of IL-6, but not TNFα, in septic mice treated with GTS-21 compared to vehicle-treated mice. Prior SPLX protected mice from developing ileus following CLP. SPLX + vehicle mice demonstrated a significantly lower serum concentration of IL-6 and TNFα compared to sham + vehicle, and colonic TNFα levels declined significantly following SPLX. Administering GTS-21 in SPLX mice did not result in an additional benefit on GI motility, nor on systemic or local colonic inflammation (Table 1).

**Table 1:** %GE, GC, serum cytokine levels (pg/ml) and colonic cytokine levels (pg/g colon) in mice that underwent splenectomy or sham surgery, followed by the CLP procedure and administration of vehicle or 8 mg/kg GTS-21

GI transit study (*n *= 11)	%GE	GC
*Nonseptic animals*	*88.96 ± 8.13*	*4.86 ± 0.45*
Sham + vehicle + CLP	53.27 ± 10.32	2.53 ± 0.42
Sham + GTS-21 8 mg/kg + CLP	83.62 ± 5.91*	4.26 ± 0.35*
SPLX + vehicle + CLP	79.49 ± 8.98*	3.86 ± 0.44*
SPLX + GTS-21 8 mg/kg + CLP	77.64 ± 11.81*	3.55 ± 0.52

**Cytokine level in serum (*n *= 8 to 11)**	**IL-6**	**TNFα**

*Nonseptic animals*	*2.57 ± 0.48*	*4.95 ± 0.29*
Sham + vehicle + CLP	243.56 ± 54.89	63.72 ± 21.25
Sham + GTS-21 8 mg/kg + CLP	90.26 ± 31.31*	54.23 ± 25.14
SPLX + vehicle + CLP	74.31 ± 17.45*	11.52 ± 1.99^#^
SPLX + GTS-21 8 mg/kg + CLP	64.98 ± 7.15*	12.81 ± 3.91^#^

**Cytokine levels in colonic tissue (*n *= 5 to 11)**	**IL-6**	**TNFα**

*Nonseptic animals*	*20.43 ± 4.87*	*41.30 ± 4.47*
Sham + vehicle + CLP	1281.85 ± 932.10	137.15 ± 61.36
Sham + GTS-21 8 mg/kg + CLP	134.88 ± 36.15	46.46 ± 5.03*
SPLX + vehicle + CLP	214.22 ± 75.88	29.97 ± 8.60*
SPLX + GTS-21 8 mg/kg + CLP	487.49 ± 343.87	46.03 ± 17.22*

Data presented as mean ± SEM. Two-way ANOVA or one-way ANOVA as appropriate with SNK *post hoc *testing; *P ≤ 0.05 versus sham + vehicle, ^#^*P *≤ 0.05 significant effect of splenectomy (SPLX).

## Conclusion

Splenectomy protects mice from developing ileus following CLP, an animal model of polymicrobial sepsis, as does the preventive administration of the selective α7nAChR agonist GTS-21. Splenectomy resulted in a reduction of systemic TNFα and IL-6 levels, indicating that the spleen is a major source of proinflammatory cytokines. GTS-21 ameliorates CLP-induced septic ileus, but did not lead to a complimentary benefit in addition to splenectomy.
